# Influence of Accelerated Carbonation on the Performance of Recycled Concrete Containing Fly Ash, Recycled Coarse Aggregate, and Fine Aggregate

**DOI:** 10.3390/ma17215191

**Published:** 2024-10-24

**Authors:** Ziqi Yao, Ling Luo, Yongjun Qin, Yu Bi, Fengchao Liu, Yiheng Yang

**Affiliations:** 1 College of Civil Engineering and Architecture, Xinjiang University, Urumqi 830017, China; 107552104185@stu.xju.edu.cn (Z.Y.); qyjjg@xju.edu.cn (Y.Q.); biyustructure@163.com (Y.B.); ikernylfc@163.com (F.L.); gtr96033@outlook.com (Y.Y.); 2 Xinjiang Civil Engineering Technology Research Center, Urumqi 830017, China

**Keywords:** recycled coarse aggregate, recycled fine aggregate, accelerated carbonation, microstructure, carbon emission

## Abstract

In order to improve the quality of solid waste utilization, this study simultaneously used recycled coarse aggregate and recycled fine aggregate to prepare recycled aggregate concrete, with fly ash partially replacing cement as a binder. After the particle gradation of recycled aggregate was artificially adjusted into continuous gradation, the effects of accelerated carbonation on the performance and microstructure of recycled concrete were studied. The microstructural change was analyzed using mercury intrusion porosimetry and scanning electron microscopy–energy dispersive spectroscopy. Additionally, the environmental benefits of the recycled concrete were evaluated based on carbon emissions using the life cycle assessment method. The experimental results indicate that accelerated carbonation can increase the compressive strength of recycled concrete by up to 13%, and its microstructure becomes more compact after carbonation. The carbon emissions are reduced by more than 13% after using 20% fly ash, contributing to sustainable development. Additionally, the optimal replacement rate of recycled fine aggregate should be controlled to under 15% when both recycled coarse and fine aggregates are used.

## 1. Introduction

In recent years, climate records have continually been broken, and the concentration of carbon dioxide (CO_2_) in the atmosphere has also reached new highs. Global temperatures are increasingly approaching the 1.5 °C warming limit specified in the Paris Agreement [1,2]. To address this situation, the Intergovernmental Panel on Climate Change has stated that countries must achieve net-zero CO_2_ emissions by 2050 [3]. China, as a major carbon emitter, proposed the ‘dual-carbon’ goals in 2020, aiming to achieve peak CO_2_ emissions by 2030 and carbon neutrality by 2060 [4]. To achieve the ‘dual-carbon’ goals, all fields should consider how to reduce carbon emissions and implement green and clean production.

Since entering the 21st century, the pace of urbanization has accelerated, with the global urbanization rate reaching 56% in 2023 [5,6]. By 2050, this rate is expected to expand to 68%, with urban populations accounting for 70% of the total population [7]. This has been accompanied by the rapid development of industries related to civil engineering and a swift increase in the consumption of construction materials. As one of the widely used building materials, concrete is extensively applied in the construction and maintenance projects for buildings and roads [8]. A large amount of sand, gravel, and ordinary Portland cement (OPC) are consumed in the concrete production process, while natural aggregate resources are also tending to be exhausted [9,10]. The OPC production process generates a significant amount of greenhouse gases that harm the environment, with approximately 0.8 to 1.0 tons of CO_2_ emitted for every ton of OPC produced [11,12]. At the same time, the amount of construction waste is increasing annually. China produces over 3 billion tons of construction and demolition waste each year, of which waste concrete accounts for 50–60% [13,14]. The traditional concrete production model can no longer meet the requirements for sustainable development.

Fly ash (FA) is one of the by-products of coal power plants. In 2021, China’s thermal power production was 5805.9 billion kW·h, resulting in the production of approximately 827 million tons of FA [15]. The land used to store FA not only incurs significant costs but also causes dust pollution and harms the ecological environment [16]. To improve this situation, it is necessary to consider recycling FA as a valuable resource [17,18]. Yao et al. studied the workability, mechanical properties, and microstructure of magnesium phosphate cement-based concrete with different contents of FA [19]. The results showed that, when the FA content exceeded 25%, the optimization effect on the pore structure of the concrete began to decline. Liang et al. evaluated the predictive performance of five algorithm models for the compressive strength of FA concrete and found that the BP-GA model yielded the best predictions [20]. Additionally, the improvement in the mechanical properties of the concrete was most effective when the FA content was below 30%. Due to the unique pozzolanic effect, morphological effect, and microaggregate effect of FA, partially replacing OPC with FA as a binder can enhance the workability, mechanical properties, and durability of concrete [21,22,23]. At the same time, it reduces CO_2_ emissions during the production of binders.

At the aggregate level, the feasibility of using demolished concrete to produce recycled aggregate has been researched. Preparing concrete with recycled aggregate instead of natural aggregate can reduce the exploitation of natural aggregate resources. Additionally, it can address the challenge of demolition and construction waste management, improve the quality of waste utilization, and promote sustainable development. Belabbas et al. used recycled coarse aggregate (RCA) from multiple recycling cycles to prepare recycled concrete at different curing temperatures (20, 40, and 60 °C) [24]. They studied its mechanical properties and established a predictive model for total shrinkage. The results indicated that multiple recycling cycles and the replacement rates of the RCA had a detrimental effect on the mechanical properties of the recycled concrete. Ma et al. studied the effects of natural fine aggregate (NFA), recycled fine aggregate (RFA), and RFA modified with sodium silicate on mortar performance, and found that the compressive strength of recycled mortar could be increased by up to about 13% when the replacement rate of the RFA was below 30% [25]. The old mortar attached to the surface of the recycled aggregate reduces the performance of recycled concrete. To discuss the effect of the residual paste content on the surface of the RFA on the performance of recycled mortar and recycled concrete, Chen et al. prepared RFA with different old paste contents using spherical glass beads to fully replace the RFA [26]. The experimental results showed that, when the residual paste content was controlled within a certain range, its adverse effects on recycled mortar and recycled concrete could be negligible. Due to the characteristics of recycled aggregate, such as a high water-absorption rate, high porosity, and the complex structure of the interface transition zone, the mechanical properties and durability of recycled aggregate concrete are inferior to those of natural aggregate concrete. However, most studies focused on the use of RCA or RFA alone, and few studies have investigated the combined use of both RCA and RFA [27,28,29,30].

It can be observed that the majority of the research focuses on recycled aggregates prepared from waste concrete generated by the demolition of buildings. In contrast, solid waste from road demolition is predominantly utilized directly for backfill in road base course and bedding course, with few applications in construction projects. This discrepancy may be attributed to the fact that the particle gradation of recycled aggregates derived from road demolition does not meet the requirements of building engineering. Additionally, the recycled aggregates exhibit a higher porosity, and the strength of the old mortar adhered to their surface is relatively low. In order to enhance the reuse value and quality of recycled aggregate prepared from road demolition waste, it is essential to investigate their potential for effective reuse in building engineering.

In addition, the service life of concrete structures is affected by a variety of factors. Among them, CO_2_ in the atmosphere accelerates the corrosion of steel rebars inside concrete structures, which is a significant cause of concrete durability failure [30]. During the OPC hydration process, pores of varying sizes are formed, allowing CO_2_ from the environment to diffuse into the concrete. This then reduces the alkalinity of the concrete [31,32]. At the same time, some studies have found that the performance of concrete improved under carbonation [33,34]. CO_2_ reacts with Ca(OH)_2_ and C-S-H, the hydration products of OPC, converting them into a stable form of CaCO_3_ [35,36]. The CaCO_3_ generated by the reaction is distributed throughout the concrete, filling the pores and improving the compactness. Life cycle assessment (LCA) is a method to evaluate the environmental impact of a product or service throughout its life cycle [11]. The carbonation reaction of concrete not only reduces the porosity of the concrete but also enables partial carbon sequestration. It is obviously a good choice to use LCA to evaluate the carbon footprints in each stage of concrete production and use. Chen et al. established a service life assessment model for carbonated reinforced concrete with supplementary cementitious materials and found that the service life could be extended from less than 20 years to more than 90 years by considering early corrosion propagation [37]. Yuan et al. reviewed the improvement in the performance of recycled concrete containing carbonated RCA and analyzed the environmental benefits of carbonated RCA through the carbon footprint, carbon accounting, and carbon intensity [38].

Therefore, this paper simultaneously used RCA and RFA produced from road demolition waste, and partially replaced OPC with FA to prepare FA recycled aggregate concrete (FARAC), aiming to investigate the effects of accelerated carbonation on the carbonation depth, unconfined compressive strength (UCS), and microstructure. Additionally, an LCA carbon emissions calculation for the FARAC was conducted to analyze the carbon emissions at various stages. This helps to adopt different carbon emission reduction measures for each stage, increasing the possibility of using FARAC in practical applications.

## 2. Materials and Experimentation

### 2.1. Materials

#### 2.1.1. Binders

**Figure 1:**
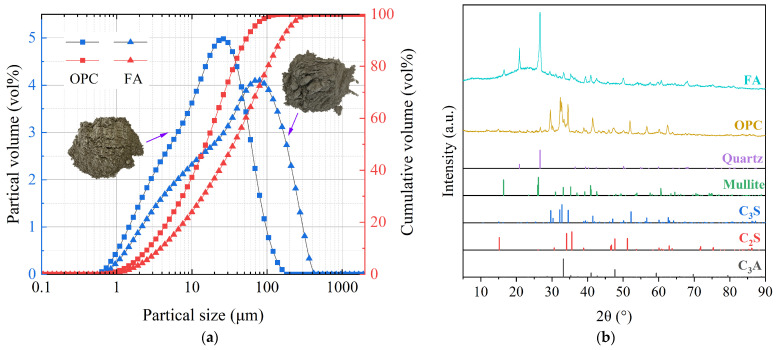
(**a**) Particle size distribution curves and (**b**) XRD patterns of the OPC and FA.

The binders used for this research consisted of Class F FA (CaO content < 10%) and OPC (P·O 42.5). The chemical compositions of the FA and OPC are shown in Table 1. The particle size distributions and X-ray diffraction (XRD) patterns of the FA and OPC are presented in Figure 1.

#### 2.1.2. Aggregates

**Figure 2:**
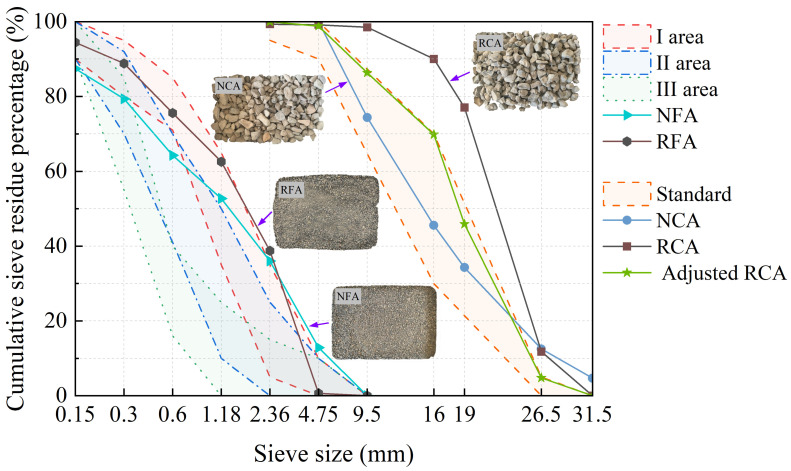
Particle size distribution of the coarse and fine aggregates.

The natural coarse aggregate (NCA) used in this paper is gravel, and the NFA is river sand. The recycled aggregates were sourced from a highway reconstruction and expansion project in the Xinjiang Uygur Autonomous Region. The physical properties of the coarse and fine aggregates were determined according to the relevant standards [39,40,41,42]. The results are shown in Table 2 and Table 3, respectively. As shown in Figure 2, the original size distribution of the RCA does not meet the requirements [42], since the coarse aggregate used in the road engineering exhibited a gap-graded distribution. Therefore, the RCA was manually re-sieved to adjust it to a continuous particle size distribution that complied with the requirements.

#### 2.1.3. Others

The water reducer was a solid polycarboxylate superplasticizer (PS) with a water reduction rate ≥ 25%. The mixing water was tap water from Urumqi.

### 2.2. Mix Design

The mix proportions for the FARAC were designed by the relevant requirements [43,44]. The experimental variables included the FA content (0, 10%, and 20%), RCA replacement rate (0, 50%, and 100%), and RFA replacement rate (0, 15%, and 30%). The mix proportions are shown in Table 4.

### 2.3. Specimen Preparation

The specimens were prepared using forced single horizontal shaft concrete mixing equipment. First, the molds (100 mm × 100 mm × 100 mm) were cleaned and a layer of oil was evenly brushed on all internal surfaces. The materials were added to the equipment in sequence and mixed. Then, the concrete mixture was placed into the pre-prepared molds and vibrated on a vibrating table until no visible large air bubbles were present on the surface. After 24 h of casting, the specimens were demolded and placed in the curing room. The temperature was controlled at (20 ± 2) °C, and the humidity was maintained at over 95%.

### 2.4. Test Methods

**Figure 3:**
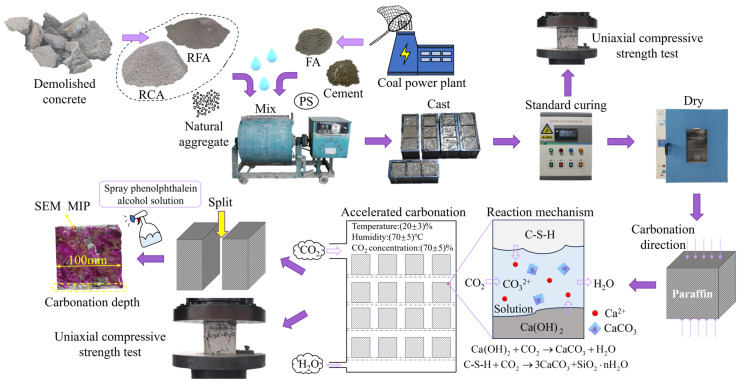
Specimen preparation and test flow chart.

The accelerated carbonation test was conducted using a CCB-70F carbonation test chamber, following the relevant requirements [45]. The compressive strength test was performed using an HCT306A microcomputer-controlled electro-hydraulic servo pressure testing machine produced by Wance Test Equipment Co., Ltd. (Shenzhen, China), according to the relevant requirements [46]. Specimens for the accelerated carbonation test were removed from the curing room after 26 days, dried for 48 h, and then had four of their surfaces sealed with paraffin, leaving two opposite faces exposed. These specimens were then placed in the carbonation test chamber for the accelerated carbonation test. The specific test procedures are shown in Figure 3.

The microstructure of the FARAC was analyzed using a mercury intrusion porosimetry (MIP) test and scanning electron microscopy–energy dispersive spectroscopy (SEM-EDS) test. The instruments used for the MIP and SEM-EDS tests were the AutoPore V-9620 mercury intrusion porosimeter produced by Micromeritics (Norcross, GA, USA) and the Sigma-300 scanning electron microscope produced by ZEISS (Oberkochen, Germany). The accelerating voltage of the SEM test was 5 kV. Samples from the compressive strength test and carbonation depth test were soaked in anhydrous ethanol for 24 h to stop hydration, and then dried and stored.

## 3. Experimental Results

### 3.1. Carbonation Depth

**Figure 4:**
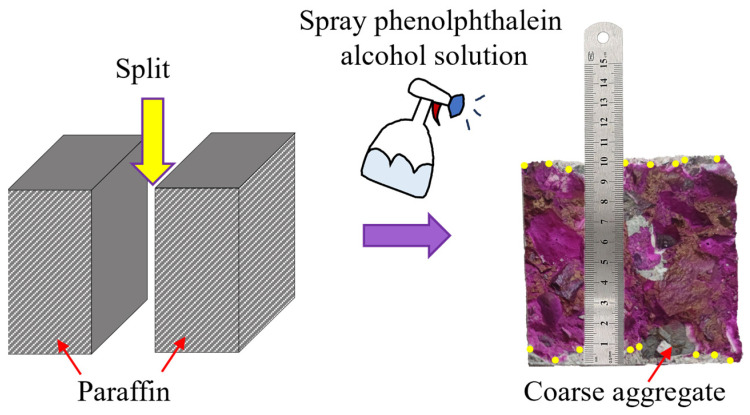
Measurement of the carbonation depth (The yellow dots are the measurement points).

**Figure 5:**
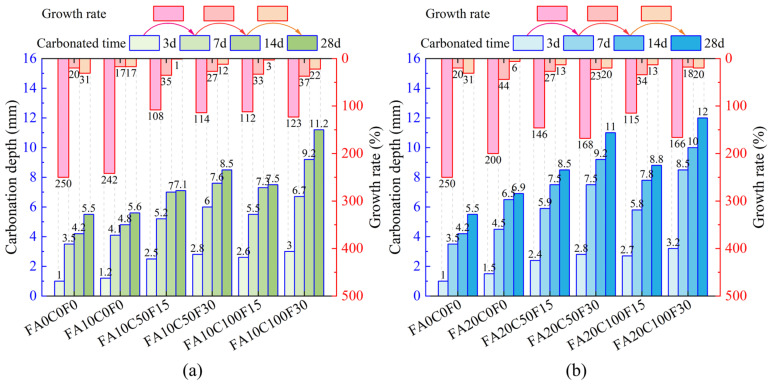
Carbonation depth and growth rate of the specimens: (**a**) FA content = 10%; (**b**) FA content = 20%.

As shown in Figure 4, after spraying the phenolphthalein solution for 30 s, a measurement point was taken at every 10 mm and the carbonation depth was measured at each point with a steel ruler. When the carbonation boundary at the measurement point coincided with a coarse aggregate particle, the arithmetic mean of the carbonation depths on both sides of the particle were taken as the carbonation depth value at that point. Finally, the arithmetic mean of the carbonation depths at all measurement points were taken as the carbonation depth of the specimen. The carbonation depths of each group of FARAC specimens were measured after 3, 7, 14, and 28 days of carbonation, and are displayed in Figure 5.

In Figure 5, the green and blue bars on the lower axis represent the carbonation depth, while the bars on the upper axis represent the growth rate. The addition of FA and recycled aggregate adversely affected the carbonation resistance of the FARAC. As the time of the carbonation increased, the carbonation depth of the FARAC continued to increase, but the rate of the increase gradually decreased.

Comparing Figure 5a and Figure 5b, the carbonation depth of the groups with the 20% FA content are always greater than that of the groups with the 10% FA content under the same replacement rates of RCA and RFA. The reason is that the incorporation of FA reduces the products of the OPC hydration reaction, which lowers the alkalinity of the concrete [47]. On the other hand, due to the insufficient hydration of FA in the early age, the porosity of the concrete is high and the carbonation resistance is reduced [48]. As the carbonation time increases, the products of the carbonation reaction and the secondary hydration products of the FA fill the internal voids of the concrete, reducing the porosity [36]. Although the FA has some beneficial effects on the carbonation resistance of the concrete, the improvement is less significant than the adverse effects, ultimately leading to an overall increase in the carbonation depth. Consistent with the findings of Chousidis et al., the addition of FA reduces the carbonation resistance of the concrete [49,50].

It can be seen that the carbonation depth of the FARAC increases with the increase in the RCA and RFA replacement rates, as shown in Figure 5. On the one hand, the permeability of the recycled aggregates is higher, and the porosity of the interface transition zone in the FARAC increases with the increase in the replacement rate of recycled aggregates [51,52]. On the other hand, the recycled concrete has also undergone carbonation in its previous service environment [50,53]. Taking 28d of carbonation as an example, in Figure 5a, the carbonation depths of FA10C50F15, FA10C50F30, FA10C100F15, and FA10C100F30 are 7.1 mm, 8.5 mm, 7.5 mm, and 11.2 mm, respectively. It is noteworthy that, when the replacement rate of the RCA is 50%, increasing the replacement rate of the RFA from 15% to 30% results in an increase in the carbonation depth by 1.4 mm. Whereas, when the replacement rate of the RFA is 15%, increasing the replacement rate of the RCA from 50% to 100% results in an increase in the carbonation depth by only 0.4 mm. The effect of the increase in the replacement rate of the RCA on the carbonation depth of the specimens is less than that of the increase in the replacement rate of the RFA. This is because the RFA has a smaller size and more old paste attached to its surface, which increases the porosity of the FARAC, making it easier for CO_2_ from the environment to enter the concrete [54]. For the groups with a 20% FA content, the same pattern is observed. From Figure 5b, after 28 days of carbonation, when the replacement rate of the RFA is 15%, and the replacement rate of the RCA increases from 50% to 100%, the carbonation depth increases from 8.5 mm to 8.8 mm, an increase of 3.5%. When the replacement rate of the RCA is 50%, and the replacement rate of the RFA increases from 15% to 30%, the carbonation depth increases from 8.5 mm to 11.0 mm, increasing by 29.4%.

### 3.2. Compressive Strength

**Figure 6:**
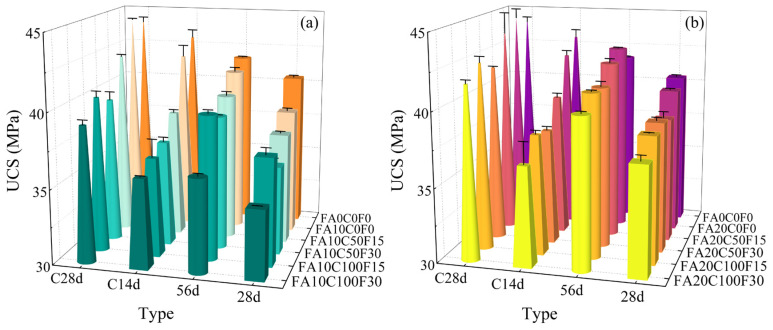
UCS results for the FARAC in the 28d, 56d, C14d, and C28d conditions: (**a**) FA content = 10%; (**b**) FA content = 20%.

The UCS of the specimens after 28 days (28d) and 56 days (56d) of standard curing, as well as after 28 days of standard curing followed by 14 days (C14d) and 28 days (C28d) of carbonation, was measured. The results are shown in Table 5 and Figure 6. It can be seen that the UCS of each group increases with the increase in the carbonation time under the same curing time. According to Table 5, the UCS results for FA0C0F0 and FA10C0F0 with 56d are 41.47 MPa and 41.07 MPa, respectively, which are smaller than the UCS results of 42.99 MPa and 41.99 MPa with C14d. In contrast, the UCS result for FA20C0F0 with 56d is 42.67 MPa, which is greater than that of 42.03 MPa with C14d. This is because, on one hand, the addition of FA reduces the generation of the OPC hydration product Ca(OH)_2_, thereby reducing the formation of the carbonation reaction product CaCO_3_. On the other hand, the FA primarily exhibits microaggregate and morphological effects during the 14 days of carbonation, which cannot compensate for the strength generated by the replaced part of the OPC. With the increase in the carbonation time, the effect of the carbonation reaction products and the secondary hydration products of the FA in filling the internal pores of the FARAC began to appear, increasing the matrix density, and thereby increasing the UCS. The UCS results for the FA10C0F0 and FA20C0F0 with 28d are 38.46 MPa and 39.09 MPa, respectively, which were both lower than those of FA0C0F0 (40.38 MPa). However, in both the 56d and C28d conditions, the UCS result for FA20C0F0 is the highest among the three groups. This indicates that the FA content of 20% has a more obvious effect on the concrete in the case of a longer reaction time.

As seen in Figure 6, the strengthening effect of the accelerated carbonation on the UCS is more pronounced for the groups using recycled aggregates. When the carbonation days increase from 14 to 28 days, the UCS of each group improves. For the groups with the 20% FA content, the UCS of FA10C50F15, FA10C50F30, FA10C100F15, and FA10C100F30 increased from 38.46 MPa, 36.99 MPa, 36.55 MPa, and 35.93 MPa to 42.21 MPa, 39.66 MPa, 40.33 MPa, and 39.05 MPa, respectively, which is an increase of 9.7%, 7.2%, 10.3%, and 8.7%. For the groups with the 20% FA content, the UCS of FA20C50F15, FA20C50F30, FA20C100F15, and FA20C100F30 increased from 39.49 MPa, 37.73 MPa, 38.04 MPa, and 36.66 MPa to 43.79 MPa, 41.84 MPa, 42.56 MPa, and 41.63 MPa, respectively, showing increases of 10.9%, 10.9%, 11.9%, and 13.6%, which are all higher than the 6.1% increase in FA20C0F0. Consistent with the previous analysis, aside from the effect of accelerated carbonation, the addition of FA begins to show a strengthening effect on the UCS in the later stages. Compared with natural aggregates, recycled aggregates have old mortar attached to their surfaces and a higher porosity, making it easier for CO_2_ to penetrate into the concrete [53,55]. This indicates that, under the same conditions, the carbonation reaction efficiency is higher in concrete where recycled aggregates replace natural aggregates, thereby improving the UCS. FA20C100F30 has the highest FA content and recycled aggregate replacement rate, so the increase in the UCS is the greatest as the carbonation time increases. However, increasing the replacement rate of the recycled aggregates also reduces the UCS of the FARAC. As shown in Figure 6b, the UCS of FA20C50F15, FA20C50F30, FA20C100F15, and FA20C100F30 with C28d decreased by 0.5%, 4.9%, 3.3%, and 5.4%, respectively, compared with FA0C0F0 (43.99 MPa). Therefore, the optimal replacement rates for the RCA and RFA are 50% and 15%, respectively.

### 3.3. Microstructure Characteristics

#### 3.3.1. MIP

**Figure 7:**
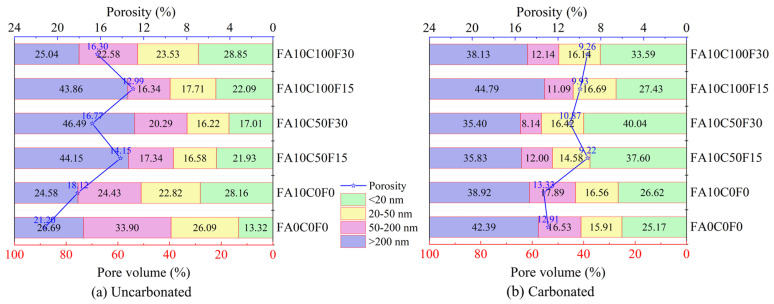
Statistics of the pore size distribution and porosity of the uncarbonated and carbonated FARAC under a 10% FA content.

A reasonable pore size distribution can improve the mechanical properties and durability of concrete. According to previous studies, the internal pores of concrete are categorized into the following four types: <20 nm (harmless pores), 20–50 nm (less harmful pores), 50–200 nm (harmful pores), and >200 nm (more harmful pores) [36,56]. To explore the effect of accelerated carbonation on the pore structure of the FARAC, six groups of uncarbonated and carbonated samples were selected for the MIP tests. The pore size percentage distributions and porosity of the uncarbonated and carbonated specimens are shown in Figure 7.

Comparing Figure 7a and Figure 7b, it is found that the porosity of each group significantly decreases after accelerated carbonation. FA0C0F0 and FA10C0F0 show reductions of 39.12% and 26.47%, respectively. As the amount of recycled aggregate increases, the reduction in the porosity of the carbonated FARAC also increases, consistent with the findings of Nguyen and Salvoldi et al. [57,58]. FA10C50F15, FA10C50F30, FA10C100F15, and FA10C100F30 decreased by 34.84%, 35.18%, 23.53%, and 43.20%, respectively. This trend is similar to the increase in the UCS of the uncarbonated and carbonated specimens as influenced by the amount of recycled aggregate.

**Figure 8:**
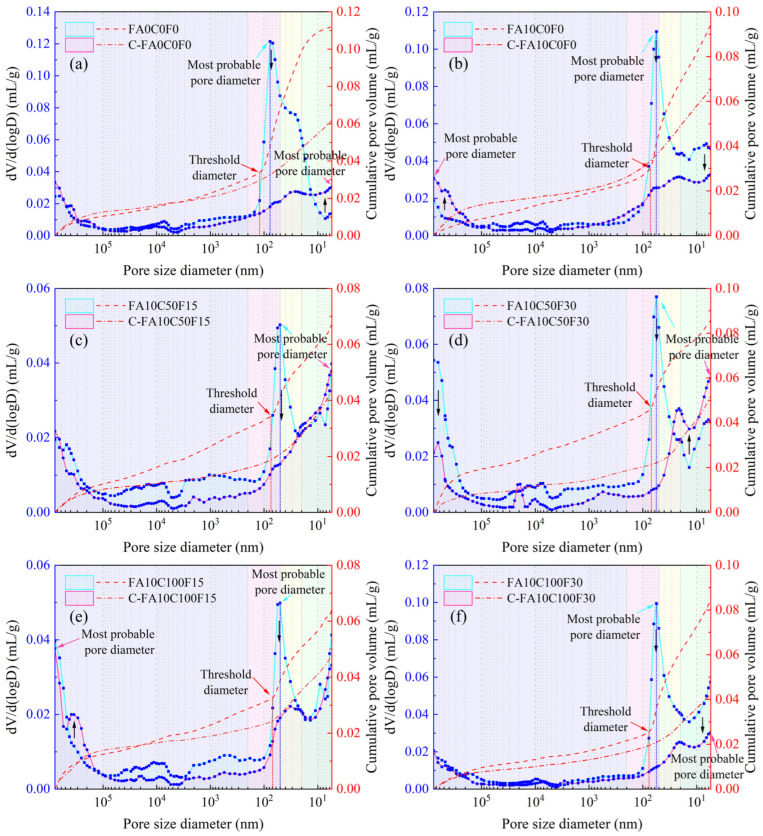
Incremental and cumulative pore volume curves of uncarbonated (group name) and carbonated (C-group name) FARAC under a 10% FA content. (**a**) FA0C0F0; (**b**) FA10C0F0; (**c**) FA10C50F15; (**d**) FA10C50F30; (**e**) FA0C100F15; (**f**) FA10C100F30.

The incremental pore volume and cumulative pore volume distribution curves of the uncarbonated and carbonated specimens for each group are shown in Figure 8. The most probable pore size refers to the pore diameter corresponding to the peak point in the incremental pore volume distribution curve, indicating the pore size with the highest frequency among all pore sizes [59]. From the blue curves in Figure 8, it can be seen that the most probable pore size of the uncarbonated specimens in each group appears in the range of 50–200 nm. As shown in Figure 7a and Figure 8a, the uncarbonated FA0C0F0 has the highest proportion of harmful pores, accounting for 33.90%. The addition of FA reduces the porosity of the concrete, corresponding to the rightward shift of the blue curves in Figure 8b–f, and, meanwhile, the peak values reduce. At this time, the FA exhibits a morphological effect, effectively dispersing OPC particles, preventing flocculation, and allowing for the full hydration of the OPC, and then increasing the structural compactness. Combining FA10C50F15 and FA10C100F15, it can be observed that, when the RFA replacement rate remains constant, increasing the RCA replacement rate has a relatively small impact on the pore distribution. However, increasing the RFA replacement rate significantly affects the pore structure of the FARAC. Similar to the results of Lian et al., from a microscopic perspective, the RCA has little effect on the stability of the concrete’s micropore structure [60]. The increase in the RFA replacement rate has a more adverse effect on the performance of the FARAC from a macroscopic perspective.

The threshold diameter refers to the inflection point of the cumulative pore volume distribution curve, which is also the maximum pore diameter of the channels connecting each pore to larger pores [61,62]. As the pressure increases, pores with diameters smaller than the threshold diameter begin to be filled, causing a surge in mercury intrusion. From Figure 8, it can be observed that the uncarbonated specimens show a distinct threshold diameter in the graph. However, the cumulative volume distribution curve of the carbonated specimens tends to be flat, making it difficult to observe the threshold diameter. This is because the accelerated carbonation results in a more uniform distribution of pores inside of the FARAC. As the carbonation progresses, CO_2_ continuously enters the concrete and reacts with Ca(OH)_2_. Various kinds of defects within the concrete, such as microvoids and microcracks, are filled with calcite crystals. These effects will ultimately lead to changes in the mechanical properties and durability of the FARAC. The relationship between the incremental pore volume and the pore size of each uncarbonated group is similar across different mix proportions, while the pore size distribution after carbonation varies significantly. The pore size distribution after carbonation shifts from a predominance of medium-sized pores to a higher proportion of larger and smaller pores, with the proportion of medium-sized pores decreasing. For most groups, the most probable pore diameter moves from the harmful pore region to the harmless pore region after carbonation. Carbonated FA10C0F0 and carbonated FA10C100F15 each have a relatively high peak in both the more harmful pore and harmless pore regions, and the two peak values are similar. The possible reason is that some of the original micro-defects are squeezed and cracked, forming larger pores or interconnected pores.

#### 3.3.2. SEM-EDS

**Figure 9:**
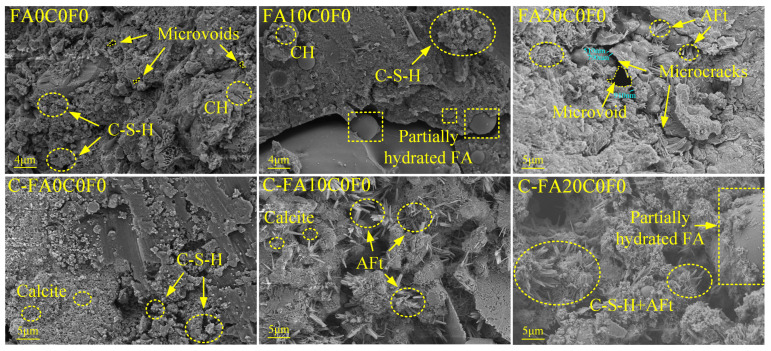
Cured 28d (Group name) and carbonated 28d (C-Group name) SEM diagrams.

**Figure 10:**
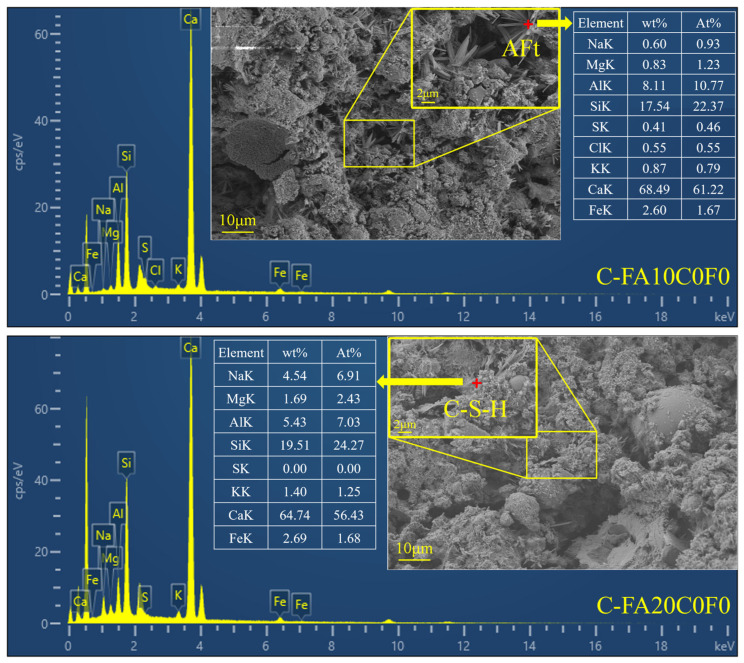
SEM-EDS diagrams of C-FA10C0F0 and C-FA20C0F0.

Since the use of recycled aggregates does not change the types of hydration products, three mix proportions of uncarbonated and carbonated specimens without recycled aggregates were selected for the SEM-EDS tests to highlight the improvement of the microstructure due to carbonation. The results are shown in Figure 9 and Figure 10. For the uncarbonated specimens, a comparison of the microstructures of FA0C0F0, FA10C0F0, and FA20C0F0 reveals that the addition of FA can improve the internal structure of the concrete. From the micrographs of FA10C0F0 and FA20C0F0, partially hydrated FA particles can be observed, indicating that FA requires a longer curing time for complete hydration, which is same as the conclusions of Nazeer et al. [63]. Comparing the micrographs of the uncarbonated and carbonated specimens in Figure 9, it is found that the quantities of calcite and ettringite increase after accelerated carbonation, making the concrete matrix denser. As depicted in Figure 10, the SEM-EDS test results reveal a notable increase in the hydration products of FA10C0F0 and FA20C0F0 after accelerated carbonation. It is observable that AFt was intertwined with C-S-H gel, which is conducive to the strength enhancement of the FARAC. This further substantiates the promotion role of FA in the hydration process. As shown in the reaction mechanism in Figure 3, during the accelerated carbonation process, CO_2_ enters the concrete through the pores and reacts with the hydration product to form calcite crystals of CaCO_3_. After that, CaCO_3_ penetrates into the microvoids and microcracks along with moisture, playing a role in pore filling [30].

## 4. Carbon Emission Analysis of FARAC

Compared with ordinary concrete, FARAC has greater environmental value. This paper quantitatively analyzes its environmental benefits using LCA carbon emissions. Zhang et al. studied the hydration reaction degree of binders with different FA contents and derived a formula for the degree of reaction of OPC and FA in relation to the w/b ratio, laying the groundwork for the calculation of carbon emissions in FA concrete [64]. Xiao et al. proposed a carbonation-absorption model for recycled concrete and conducted an environmental assessment based on LCA [65]. Based on the carbon emission calculation model by Xiao et al., this paper analyzed the LCA carbon emissions of FARAC by integrating the existing carbon emission evaluation system of recycled concrete.

The total carbon emissions *C_T_* and LCA carbon emissions *C_L_* were calculated according to Equations (1) and (2), respectively, where *C_i_* represents the carbon emissions at each stage; the calculation formulae can be found in Table 6.

(1) $$C_{T}=C_{1}+C_{2}+C_{3}+C_{4}+C_{6}$$

(2) $$C_{L}=C_{T}-C_{5}$$

In Equation (8), *m*_0_ is the amount of CO_2_ absorbed when 1 m^3^ of FARAC is fully carbonated, calculated according to Equation (11) [66], as follows:

(11) $$m_{0}=\left(1-\beta_{f}\right)6.99d-4\gamma \frac{{\mathrm{Al}}_{2}O_{3}\%}{102}\beta_{f}d\times 10^{3}$$

where

Al_2_O_3_%—the Al_2_O_3_ content (%) in the FA. Al_2_O_3_% is 21.08% for this research.
*β_f_*—the FA content (%).
*d*—the total amount (kg/m^3^) of binders in 1 m^3^ of FARAC; *d* is 487.5 kg/m^3^ for this research.

*γ*—the pozzolanic reaction degree coefficient of the FA. It is 0.2 before 28 days of curing, and calculated using interpolation after 28 days of curing [64]. For a *w*/*b* ratio of 0.4, *γ* is taken as 0.45 for this research.

*x_c_* is the carbonation depth of the FARAC when fully carbonated and *A_surface_* is the exposed surface area of 1 m^3^ of the FARAC in use. *x_c_* is calculated according to Equation (12), and *A_surface_* is taken as 5.68 m^2^ [65,67], as follows:

(12) $$x_{c}=839g_{\mathrm{RCA}}{\left(1-R\right)}^{1.1}\sqrt{\frac{\left(W/\left(\gamma_{c}C\right)-0.34\right)}{\gamma_{\mathrm{HD}}\gamma_{c}\times 8.03C}n_{0}t}$$

where

*R*—the relative humidity (%). *R* is taken as the annual average relative humidity of Urumqi for this research, which is 57.5%.
*W*—the amount of mixing water (kg); *W* is 195 kg for this research.
*γ_c_*—the OPC correction factor. It can be found in Table 7.
*C*—the amount (kg) of OPC. It can be found in Table 7.
*γ_HD_*—the OPC hydration correction factor; *γ_HD_* is taken as 0.85 for 28 days of curing.
*n*_0_—the volume fraction (%) of CO_2_; *n*_0_ is taken as 0.03%.
*t*—the carbonation time (day); *t* is taken as 50 years for the calculation of the designed service life for this research.
*g_RCA_*—the influence coefficient of the RCA; *g_RCA_* is taken by linear interpolation, ranging from 1 to 1.5 as the replacement rate of the RCA increases from 0 to 100%.

**Table 7:** Parameters related to *m*_0_ and *x_c_*.

Group	*β_f_*	*g_RCA_*	*g_RFA_*	*γ_c_*	*C*	*m*_0_	*x_c_*
	(%)	-	-	-	(kg)	(kg)	(mm)
FA0C0F0	0	1	1	1	488	3408	3.25
FA10C0F0	10	1	1	0.9	439	2886	5.79
FA10C50F15	10	1.25	1.25	0.9	439	2886	9.04
FA10C50F30	10	1.25	1.5	0.9	439	2886	10.85
FA10C100F15	10	1.5	1.25	0.9	439	2886	10.85
FA10C100F30	10	1.5	1.5	0.9	439	2886	13.02
FA20C0F0	20	1	1	0.8	390	2363	8.86
FA20C50F15	20	1.25	1.25	0.8	390	2363	13.84
FA20C50F30	20	1.25	1.5	0.8	390	2363	16.61
FA20C100F15	20	1.5	1.25	0.8	390	2363	16.61
FA20C100F30	20	1.5	1.5	0.8	390	2363	19.94

From the experimental results mentioned earlier, it is evident that the RFA has a more significant impact on the carbonation resistance of the FARAC compared with the RCA. Therefore, based on Equation (12), the influence coefficient for RFA *g_RFA_* is added, resulting in Equation (13). When the replacement rate of the RFA is zero, *g_RFA_* is taken as one, and when the replacement rate is 30%, *g_RFA_* is taken as one and five-tenths. For the intermediate replacement rates, the value is determined by linear interpolation. The calculations for *m*_0_, *x_c_*, and related parameters can be found in Table 7.

(13) $$x_{c}=839g_{\mathrm{RCA}}g_{\mathrm{RFA}}{\left(1-R\right)}^{1.1}\sqrt{\frac{\left(W/\left(\gamma_{c}C\right)-0.34\right)}{\gamma_{\mathrm{HD}}\gamma_{c}\times 8.03C}n_{0}t}$$

**Figure 11:**
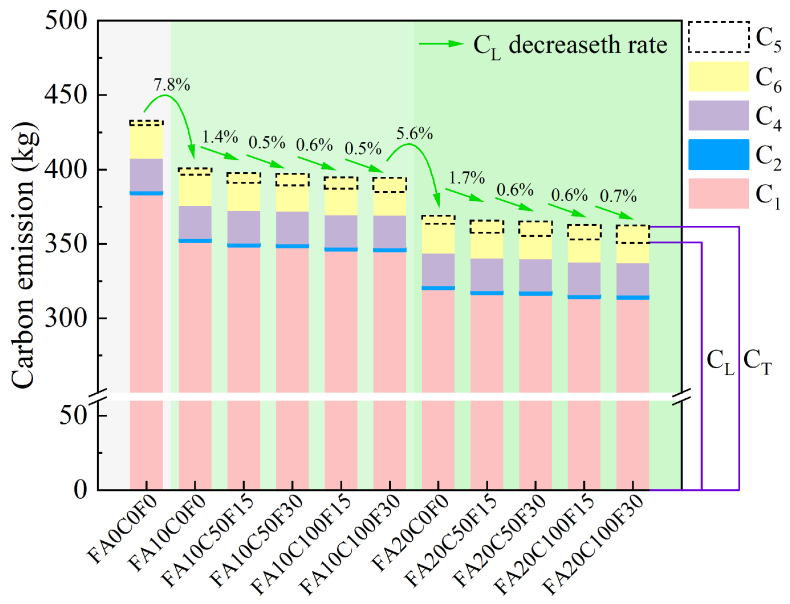
Carbon emissions of 1 m^3^ of FARAC.

Considering Equation (6), the average carbon emissions per m^3^ for the three main components—beams, slabs, and columns—and an approximately 2% loss during the mixing process, *C*_4_, is taken as 21.8 kg [65,68]. FARAC is used on-site after curing in this research, so *C*_3_ is zero. The LCA carbon emissions of 1 m^3^ of FARAC are shown in Table 8 and Figure 11 [69,70].

As shown in Table 8, the LCA carbon emissions of the FARAC with different mix proportions decrease with the increase in the FA content and the replacement rate of the recycled aggregate. Compared with FA0C0F0, the *C_L_* of FA10C0F0 and FA20C0F0 decreased by 7.8% and 15.46%, respectively. It can also be seen that the largest proportion of *C_T_* comes from *C*_1*a*_ and *C*_1*b*_. This is consistent with the research results of Xia and Xiao et al. [71,72]. The production of OPC generates a large amount of carbon emissions, and the use of supplementary cementitious materials such as FA and slag to partially replace OPC can reduce the carbon emissions from raw material production, thereby effectively reducing the carbon emissions of concrete [11].

It can be seen from Figure 11 that, with the increase in the replacement rate of the recycled aggregate, *C*_5_ gradually increases. The high porosity and surface-attached mortar of recycled aggregates make them more favorable for carbon sequestration compared with natural aggregates. Additionally, the performance of the recycled aggregates is enhanced after carbonation, promoting green and sustainable development [14,55]. However, it is necessary to consider the problem of concrete becoming more neutral during the accelerated carbonation process. In actual service scenarios, the reduction in the pH will accelerate the corrosion of the internal steel rebars, which is detrimental to the durability of the structure.

## 5. Conclusions

The carbonation resistance and post-carbonation performance improvement under the accelerated carbonation of FARAC were studied. The effects of using FA, RCA, and RFA were analyzed using the carbonation depth and compressive strength as indicators, and the environmental benefits of FARAC were evaluated in conjunction with carbon emission analysis. The main conclusions can be drawn as follows:

(1) With the increase in the FA content and the replacement rate of the recycled aggregate, the carbonation depth of the FARAC gradually increases. After 28 days of carbonation, the carbonation depth of FA20C100F30 increased by 118% compared with FA0C0F0. As the carbonation time increases, the growth rate of the carbonation depth decreases, and the carbonation resistance of the FARAC improves in the later stages of the reaction.

(2) The accelerated carbonation increases the compressive strength of the FARAC. The higher the FA content and recycled aggregate replacement rate are, more obvious is the strengthening effect. The compressive strength of FA20C50F15, FA20C50F30, FA20C100F15, and FA20C100F30 increased by 8.94%, 13.92%, 7.84%, 10.43%, and 11.31% after 28 days of carbonation.

(3) From a microscopic perspective, accelerated carbonation improves the internal structure of the FARAC. After carbonation, the porosity of each group is significantly reduced, and the pore size of the most probable pore diameter becomes smaller. According to the SEM-EDS images, the concrete matrix becomes denser after carbonation.

(4) The use of FA and recycled aggregate reduces the carbon emissions at this stage. Compared with ordinary concrete, the LCA carbon emissions of FA20C50F15 decreased by 16.9%. Considering the performance and environmental benefits, the optimal mix proportion of FARAC is a 20% FA content, a 50% replacement rate of RCA, and a 15% replacement rate of RFA.

According to the research, the solid waste generated from road demolition can be effectively utilized in construction engineering. The use of FARAC can both reduce the consumption of natural resources and decrease CO_2_ emissions, offering significant environmental, economic, and social benefits. To broaden the application scenarios of FARAC, further in-depth research is needed. The mechanical properties, durability, and microstructure of FARAC under the coupling of various adverse factors require further investigation. In addition, with the development of technology, research on FARAC can be integrated with artificial intelligence, intelligent construction, machine learning, and other methods to demonstrate more potential capabilities.

## Figures and Tables

**Table 1:** Main chemical compositions of the binders (wt/%).

Binders	CaO	SiO_2_	Al_2_O_3_	Fe_2_O_3_	SO_3_	MgO	Na_2_O	LOI
OPC	52.77	23.40	6.82	4.44	4.06	2.46	1.14	2.28
FA	5.63	52.99	21.08	5.33	1.13	1.66	1.27	6.80

**Table 2:** Physical properties of the coarse aggregates.

Type	Bulk Density	Apparent Density	Elongated and Flaky Particle Content	Crushing Index	Clay Content	Water Absorption
	(kg/m^3^)	(kg/m^3^)	(%)	(%)	(%)	(%)
NCA	1572	2588	1.8	3.5	0.8	1.1
RCA	1429	2469	4.4	8.2	0.9	4.2

**Table 3:** Physical properties of the fine aggregates.

Type	Fineness Module	Bulk Density	Apparent Density	Porosity	Clay Content
		(kg/m^3^)	(kg/m^3^)	(%)	(%)
NFA	2.6	1581	2577	38.65	0.80
RFA	2.6	1468	2552	40.27	0.85

**Table 4:** Mix proportions of the FARAC.

Group	w/b	OPC	FA	NCA	RCA	NFA	RFA	Water	PS
		(kg/m^3^)	(kg/m^3^)	(kg/m^3^)	(kg/m^3^)	(kg/m^3^)	(kg/m^3^)	(kg/m^3^)	(%)
FA0C0F0 *	0.4	488	0	1181	0	636	0	195	1
FA10C0F0	0.4	439	49	1181	0	636	0	195	1
FA10C50F15	0.4	439	49	591	591	541	95	195	1
FA10C50F30	0.4	439	49	591	591	445	191	195	1
FA10C100F15	0.4	439	49	0	1181	541	95	195	1
FA10C100F30	0.4	439	49	0	1181	445	191	195	1
FA20C0F0	0.4	390	98	1181	0	636	0	195	1
FA20C50F15	0.4	390	98	591	591	541	95	195	1
FA20C50F30	0.4	390	98	591	591	445	191	195	1
FA20C100F15	0.4	390	98	0	1181	541	95	195	1
FA20C100F30	0.4	390	98	0	1181	445	191	195	1

* C represents RCA, F represents RFA, and the numbers represent the content of FA and the replacement rates of RCA and RFA.

**Table 5:** UCS results for the FARAC in the 28d, 56d, C14d, and C28d conditions.

Group	UCS (MPa)			
	28d	56d	C14d	C28d
FA0C0F0	40.38	41.67	42.99	43.99
FA10C0F0	38.46	41.07	41.99	44.47
FA10C50F15	37.44	39.85	38.46	42.21
FA10C50F30	35.91	38.99	36.99	39.66
FA10C100F15	37.22	39.65	36.55	40.33
FA10C100F30	34.64	36.26	35.93	39.05
FA20C0F0	39.90	42.67	42.03	44.58
FA20C50F15	38.44	42.05	39.49	43.79
FA20C50F30	38.80	40.88	37.73	41.84
FA20C100F15	38.54	41.03	38.04	42.56
FA20C100F30	37.40	40.14	36.66	41.63

**Table 6:** Calculation formulae for LCA carbon emissions.

C_i_	Source	Formula		
i = 1	Raw materials	$C_{1}=C_{1a}+C_{1b}$	(3)	*i*—Type of raw material. *j*—Type of energy consumption. *y*—Mode of transport. *a_ij_*—*j* energy consumption in the material *i* production process. *K_j_*—Carbon emission factor for *j* energy. *m_i_*—Amount of material *i* in 1 m^3^ of FARAC. *g_i_*—Carbon emissions generated by material *i* in the OPC production process. *d^y^*—Direct carbon emission factor using the *y* transport mode. $b_{j}^{y}$—Specific energy consumption of the *y* transport mode. *k_j_′*—Indirect carbon emission factor for *j* energy. *s_i_*—Transportation distance of material *i.* *e_j_*—*j* energy consumption in the production process of 1 m^3^ of FARAC. *s_c_*—Transportation distance of FARAC. *M*—Total mass of 1 m^3^ of FARAC.
	Production of raw materials	$C_{1a}=\sum_{i}\left(\sum_{j}a_{ij}K_{j}\right)m_{i}+\sum_{i}g_{i}m_{i}$	(4)	
	Transportation of raw materials to the mixing plant	$C_{1b}=\sum_{i}\left(d^{y}+b_{j}^{y}k_{j}^{\prime }\right)s_{i}m_{i}$	(5)	
i = 2	Production of FARAC	$C_{2}=\sum_{j}e_{j}K_{j}$	(6)	
i = 3	Transportation of FARAC to the usage site	$C_{3}=\left(d^{y}+b_{j}^{y}k_{j}^{\prime }\right)s_{c}M$	(7)	
i = 4	Construction	C_4_ takes the average carbon emissions of each major component.		
i = 5	The amount of CO_2_ absorbed by FARAC during carbonation	$\begin{array}{cc} C_{5} & =0.044m_{0}\frac{V_{c}}{V_{0}}\\ & =0.044m_{0}\frac{x_{c}A_{\mathrm{surface}}}{1} \end{array}$	(8)	
i = 6	Disposal of FARAC	$C_{6}=C_{6a}+C_{6b}$	(9)	
	Demolition of FARAC	$C_{6a}=0.9C_{4}$	(10)	
	Transportation of discarded FARAC	C_6b_ is calculated with reference to C_3_.		

**Table 8:** LCA carbon emissions of 1 m^3^ of FARAC (kg).

Group	*C*_1_		*C*_2_	*C*_3_	*C*_4_	*C*_5_	*C*_6_		*C_T_*	*C_L_*
	*C*_1*a*_	*C*_1*b*_					*C*_6*a*_	*C*_6*b*_		
FA0C0F0	313.0	70.4	2.4	0	21.8	2.8	19.6	5.2	432.4	429.6
FA10C0F0	282.0	69.4	2.4	0	21.8	4.2	19.6	5.2	400.4	396.2
FA10C50F15	285.1	63.1	2.4	0	21.8	6.5	19.6	5.2	397.2	390.7
FA10C50F30	285.5	62.2	2.4	0	21.8	7.8	19.6	5.2	396.7	388.9
FA10C100F15	287.8	57.7	2.4	0	21.8	7.8	19.6	5.2	394.5	386.7
FA10C100F30	288.2	56.8	2.4	0	21.8	9.4	19.6	5.2	394.0	384.6
FA20C0F0	250.9	68.5	2.4	0	21.8	5.2	19.6	5.2	368.5	363.2
FA20C50F15	254.1	62.2	2.4	0	21.8	8.2	19.6	5.2	365.3	357.1
FA20C50F30	254.5	61.3	2.4	0	21.8	9.8	19.6	5.2	364.8	355.0
FA20C100F15	256.8	56.8	2.4	0	21.8	9.8	19.6	5.2	362.5	352.7
FA20C100F30	257.2	55.9	2.4	0	21.8	11.8	19.6	5.2	362.1	350.3

## Data Availability

All data generated or analyzed during this study are included in this published article.
